# An updated systemic review and meta-analysis on human papillomavirus in breast carcinogenesis

**DOI:** 10.3389/fonc.2023.1219161

**Published:** 2023-08-11

**Authors:** Usman Ayub Awan, Aamer Ali Khattak, Noman Ahmed, Xingyi Guo, Sohail Akhtar, Shehrish Kamran, Zhao Yongjing, Jianbo Liu, Suliman Khan

**Affiliations:** ^1^ Medical Research Center, The Second Affiliated Hospital of Zhengzhou University, Zhengzhou, China; ^2^ Department of Medical Laboratory Technology, The University of Haripur, Haripur, Khyber Pakhtunkhwa, Pakistan; ^3^ Division of Epidemiology, Department of Medicine, Vanderbilt Epidemiology Center, Vanderbilt-Ingram Cancer Center, Vanderbilt University School of Medicine, Nashville, TN, United States; ^4^ Department of Biomedical Informatics, Vanderbilt University School of Medicine, Nashville, TN, United States; ^5^ Department of Mathematics and Statistics, The University of Haripur, Haripur, Khyber Pakhtunkhwa, Pakistan; ^6^ Department of Pathology, Shaukat Khanum Memorial Cancer Hospital and Research Center (SKMCH&RC), Lahore, Pakistan; ^7^ Zhengzhou Key Laboratory of Children’s Infection and Immunity, Children’s Hospital Affiliated to Zhengzhou University, Zhengzhou, China; ^8^ Henan Key Laboratory of Precision Diagnosis of Respiratory Infectious Diseases, Zhengzhou Key Laboratory of Precision Diagnosis of Respiratory Infectious Diseases, The Second Affiliated Hospital of Zhengzhou University, Zhengzhou, Henan, China

**Keywords:** human papillomavirus, breast cancer, viral oncology, HPV subtypes, breast carcinogenesis, meta-analysis, polymerase chain reaction (PCR), case-control studies

## Abstract

**Background:**

Breast Cancer (BC) stands out as the widely prevalent malignancy among all the types of cancer affecting women worldwide. There is significant evidence that the pathogenicity of BC may be altered by Human Papillomavirus (HPV) infection; however, conclusive data are not yet available.

**Methods:**

By searching five databases, including EMBASE, IBECS, PubMed, Scopus, Science Direct, Google Scholar, and Web of Science, a thorough systematic analysis was conducted on the prevalence of HPV in BC patients from 1990 to June 30, 2022. After applying extensive eligibility criteria, we selected 74 publications for further analysis based on the prevalence of HPV infections in breast tissues. All of the data were analyzed using a random-effects meta-analysis, Cochran Q test and *I^2^* statistic were used to calculate the heterogeneity of the prevalence among these studies using subgroup analysis. Variations in the HPV prevalence estimates in different subgroups were evaluated by subgroup meta-analysis.

**Results:**

In total, 3156 studies were initially screened, resulting in 93 full-text studies reviewed, with 74 meeting inclusion criteria. Among a total of 7156 BC biopsies, the pool prevalence of HPV was 25.6% (95% CI= 0.24-0.33, τ2 = 0.0369 with significant heterogeneity between estimates (*I* ^2 = ^97% and *p*< 0.01). Consequently, 45 studies with available controls were further studied, and the prevalence of HPV in case-control studies was 26.2% with overall odds 5.55 (95% CI= 3.67-8.41, *I* ^2 = ^38%, τ2 = 1.4878, *p*< 0.01). Further subgroup analysis of HPV revealed HPV-16 had a maximum prevalence of 9.6% (95% CI= 3.06-11.86, *I* ^2 = ^0%, τ2 = 0.6111, *p<* 0.01). Among different geographical regions, Europe reported the maximum prevalence of HPV, i.e., 39.2% (95% CI=1.29-7.91, *I* ^2 = ^18%, τ2 = 1.2911, *p*< 0.01). Overall distribution showed HPV-18 was a frequent HPV subtype reported in Australia.

**Conclusion:**

Current study provides a global estimate of HPV prevalence in BC patients and demonstrates a significant association between this virus and BC etiology. Nevertheless, we recommend further investigation into the underlying mechanism is essential to validate this hypothesis.

## 1 Introduction

Breast Cancer (BC) is the most frequently detected in women, accounting for the sixth highest cancer mortality rate and the most frequent cancer in females worldwide (1, 2). Besides, this malignancy has a substantially higher fatality rate than lung and colorectal cancers (2, 3). BC is attributed to the lives of one woman every minute and over 1,400 women every day. Recent estimates show BC affected 2.3 million women worldwide in 2020, resulting in about 0.68 million fatalities (4). Alarmingly, if the problem is not addressed, new cases might reach 2.7 million by 2030, with a death toll of 0.87 million (5). In the recent two decades, the global BC incidence has grown alarmingly, attributed to an upsurge in identified and/or undeclared risk factors. However, some risk factors may be important oncogenic infectious agents (6–8). BC is caused by several etiological variables, such as prolonged or excessive estrogen exposure brought on by early menarche (under 12 years), late menopause (> fifty-five years), increased exposure to radiation, nulliparity, alcohol addiction, and fat-rich diet (9). Amongst different factors, BC has been strongly linked to viral infection; these factors ranked between 20 and 50% of BC cases (10–12). Studies have suggested that oncoviruses, notably the Human Papillomavirus (HPV), may be risk factors for breast carcinogenesis (13). Additionally, it has been suggested that the development of BC may be influenced by the Epstein-Barr virus (EBV), the Mouse Mammary Tumor Virus (MMTV), and the Bovine Leukemia Virus (BLV) (14).

HPV is a non-enveloped double-stranded DNA virus whose genome is divided into three regions: the long control region (LCR), the early region (which encodes E1, E2, and E4-E7), and the late region (encodes L1 and L2) (15–17). E6 and E7 oncoproteins largely promote host cellular proliferation (18). The carcinogenic risk of HPVs is used to categorize subtypes into either Low-Risk (LR) or High-Risk (HR) subtypes (19). Approximately 15 HPV subtypes have been identified as HR-HPVs (16, 18, 31, 33, 35, 39, 45, 51, 52, 56, 58, 59, 68, 73, and 82) (20, 21), with their relative carcinogenic risk assessed from a variety of epidemiological studies (21). LR-HPVs typically result in anogenital and cutaneous warts, whereas HR-HPVs are responsible for approximately 4.5% of all diagnosed human malignancies, including head and neck cancers (such as oral, tonsil, and throat cancers) as well as anogenital cancers (such as cervical, anal, vulvar, vaginal, and penile cancers) (14, 22–26).

**Figure 1:**
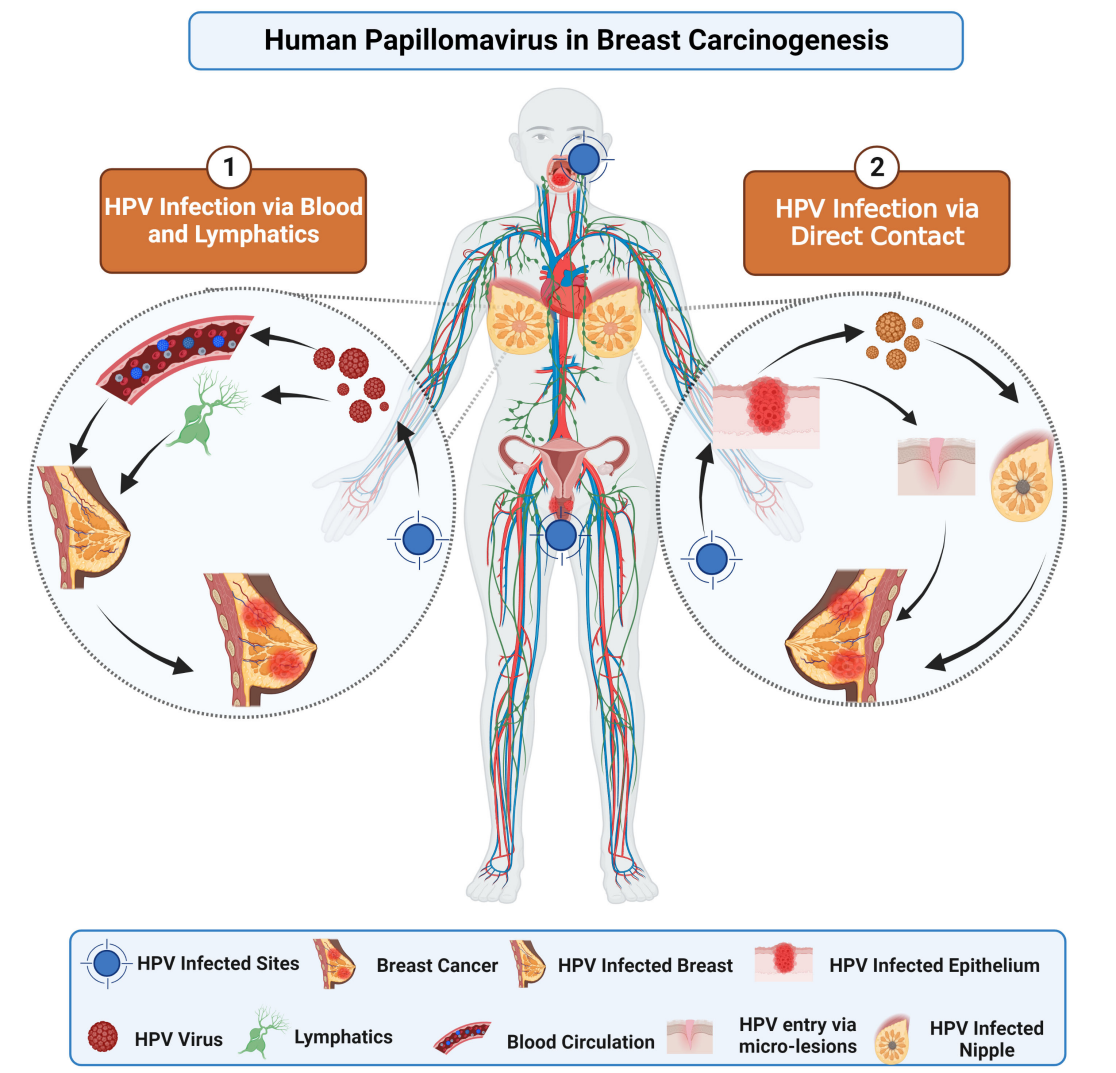
This image depicts the potential pathways implicated in BC pathogenesis. The oncogenic proteins of HPV, E6 and E7, interfere with different pathways by inactivating tumor suppressor genes. Consequently, overexpression of E6 and E7 may contribute to BC progression.

The transmission of HPV can happen in either sexual or nonsexual interactions. Direct skin-to-skin contact during sexual activity with a person infected with the virus is the most prevalent way for genital HPV to spread (27). It is suggested that HPV may enter the body through various routes, including cuts, scrapes, and mucosal membranes, and can cause infection in the stratum basale’s—the lowest layer of the stratified epithelium (27). Multiple routes exist for transferring HPV from infected sites to breast tissues, as depicted in **Figure 1**. First, HPV virions can spread to the breasts from other body parts (most commonly the head, neck, or cervical region) via the circulatory or lymphatic systems (28, 29). Second, the transmission involves the transfer of HPV to the breast through genital-breast sexual activity-related nipple or micro-lesions in the breast skin (27, 30).

In BC patients, the rate of HPV infection is anywhere between 0% to 86.2% (31); however, certain researchers have not found any association with HPV in breast carcinogenesis (32–34). The epidemiology of HPV types varies among different populations. Other investigations have discovered that the pathogenicity of HPV is determined by its genotypes (35). HPV16, HPV18, and HPV33 were frequently reported and responsible for 70% of BC cases globally (32, 36). There is currently a debate surrounding studies investigating HPV prevalence in BC. The first association between HPV and BC was first reported by, Lonardo and colleagues in 1992 (37). In the last several decades, multiple studies have highlighted the presence of HPV DNA in BC cells (2, 31, 38); certain studies have reported the absence of HPV DNA in BC tissues (39–42).

Though the involvement of HPV in breast carcinogenesis is still debatable, understanding it is essential in devising effective new preventative and treatment measures for women with BC. Consequently, the present systematic review and meta-analysis aim to address this information gap by providing an up-to-date estimate of the overall prevalence of HPV and its subtypes in BC patients worldwide.

## 2 Methodology

### 2.1 Search strategy

We systematically analyzed publications on HPV prevalence in BC patients published between 1990 and June 30, 2022. We used five databases: EMBASE, IBECS, PubMed, Scopus, Science Direct, Google Scholar, and Web of Science. Different keywords or MeSH terms were used to find relevant data, with the articles limited written in English (**Supplementary File**).

### 2.2 Eligibility criteria

By screening the title, reading the abstract, and reviewing the publications, the inclusion criteria guaranteed that the study was relevant. All cross-sectional, case-based, and prevalence-based research was also included. The following studies, on the other hand, were excluded: (i) studies published in a language other than English, (ii) studies composed of letters, commentaries, reviews, series, editorial, and commission, (iii) duplicate studies, and (iv) non-relevant studies that did not report any correlation between HPV and BC.

### 2.3 Data extraction

**Figure 2:**
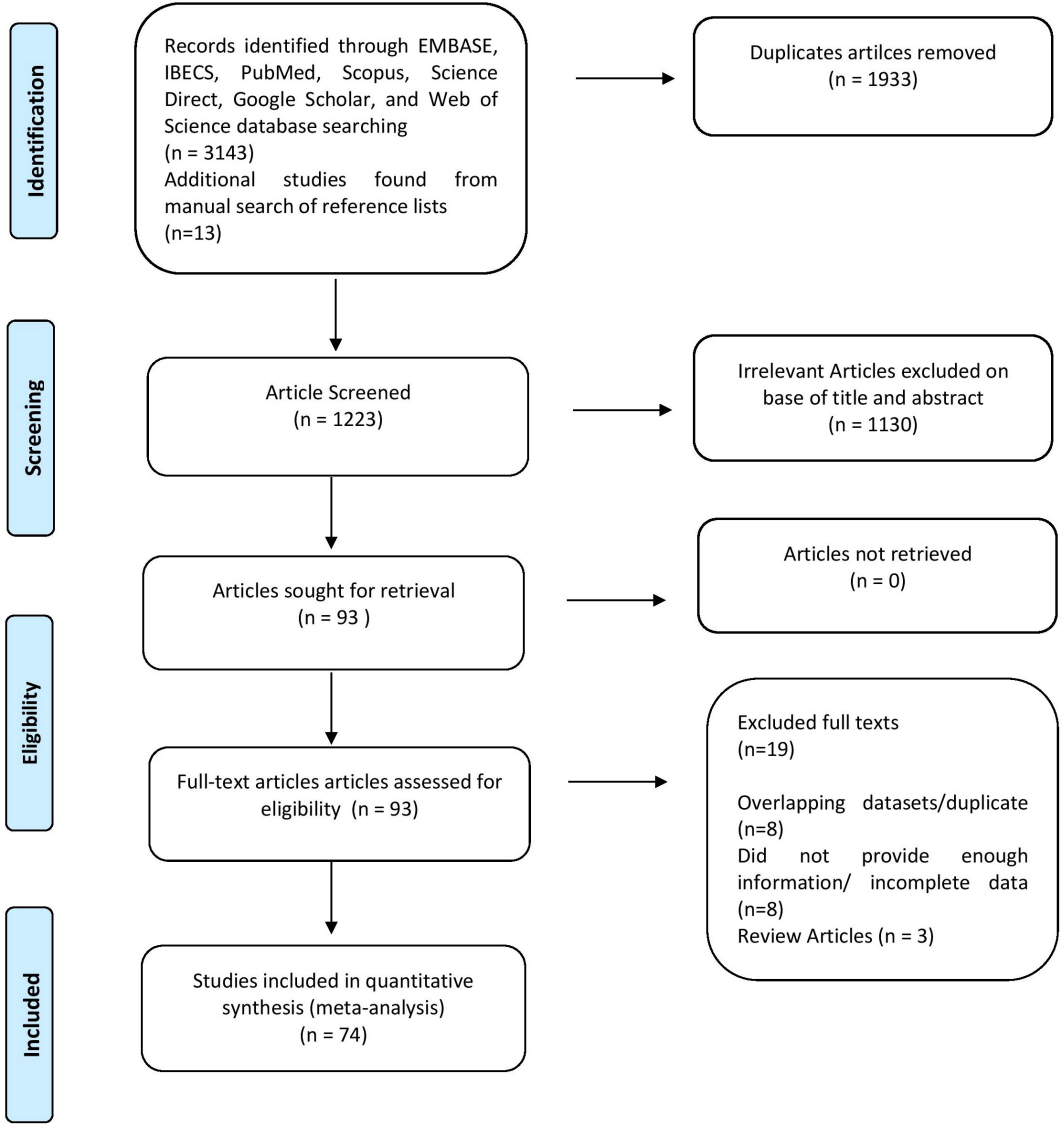
PRISMA Checklist used for paper screening in this meta-analysis.

Following selecting relevant publications, three reviewers (U.A.A., A.A.K., and N.A.) independently reviewed the titles and abstracts of articles to be read in full text. Within three decades, 3156 studies were researched using electronic databases. After a thorough evaluation, 74 pertinent papers were included. Information about the author(s), year of publication, area, sample size, prevalence of HPV in BC, HPV subtypes, coinfection frequency, and detection technique was acquired for each. Results were classified following the Preferred Reporting Items for Systematic and Meta-analyses (PRISMA) guideline, as shown in **Figure 2**.

### 2.4 Quality assessment of studies

Additionally, using the Quality Assessment Tool for Observational Cohort and Cross-Sectional Studies, three reviewers (U.A.A, A.A.K., and N.A) independently assessed the methodological quality of each included study (43). Disagreements about the quality assessment standards were settled by discussion and consensus with other study authors.

### 2.5 Statistical analysis

After extracting the data using Microsoft Excel, we conducted a statistical analysis using R version (4.2.3) for meta-analysis using different packages. HPV infection prevalence was determined using the binomial distribution, p^^=xn and the standard error $\left(se=\sqrt{\frac{\hat{p}\hat{q}}{n}}\right)$ for each study was computed. We use random-effects models to adjust for the high heterogeneity of the studies. Otherwise, it generates study impediments, mostly used to distinguish between unique research variants. We assessed the heterogeneity among the studies using the Cochran Q test and *I* ^2^ statistic, and we presented the results graphically using forest plots (showing the effect size [ES] with a 95% confidence interval [95% CI]). Therefore, the *I* ^2^ statistic, which ranges from 0 to 100%, reveals that heterogeneity, rather than chance, is the primary difference across research in systematic reviews and meta-analyses. *I^2^* values of 25%, 50%, and 70% indicate low, medium, and high trial heterogeneity. However, subgroup meta-analysis were used to identify the potential sources of statistical heterogeneity.

## 3 Results

In this systematic review and meta-analysis, we found 3156 studies after a baseline online search between 1992 and June 30, 2022, whereas 06 were discovered after manually searching reference lists, as shown in **Supplementary Table 1**. Following the exclusion of 1933 duplicate research, a total of 1223 papers were evaluated. In addition, 1130 of the articles were eliminated for being irrelevant. The remaining 93 pieces of research were extensively evaluated, and 19 full-text publications were removed owing to disparities in the nature and outcomes of investigations. In the end, only 74 studies met the inclusion criteria, and data were retrieved for the meta-analysis. A detailed PRISMA diagram depicting the publication trajectory of this meta-analysis is given in **Figure 2**.

**Figure 3:**
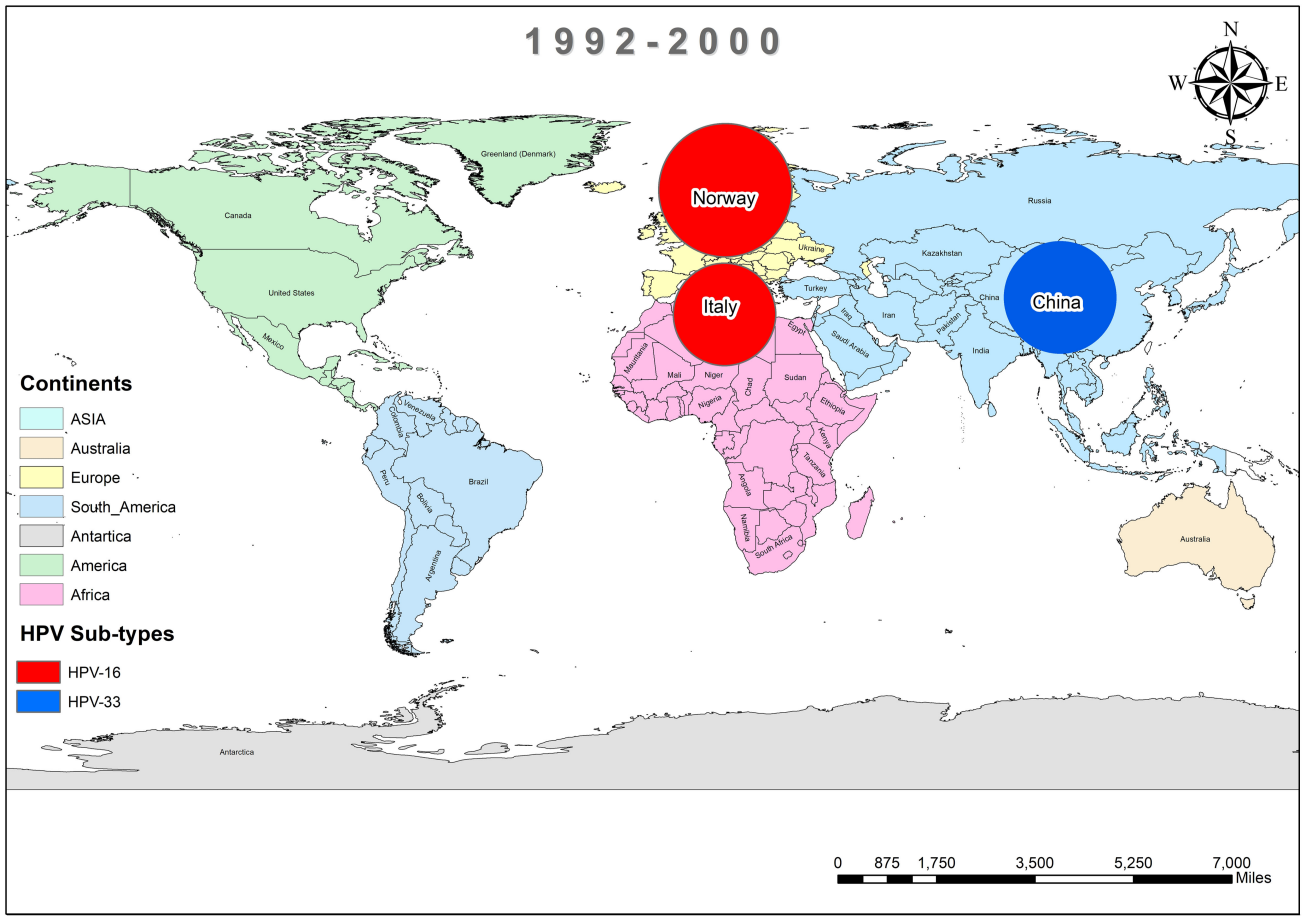
Pool prevalence of HPV in BC patients from 1990-2000.

**Figure 4:**
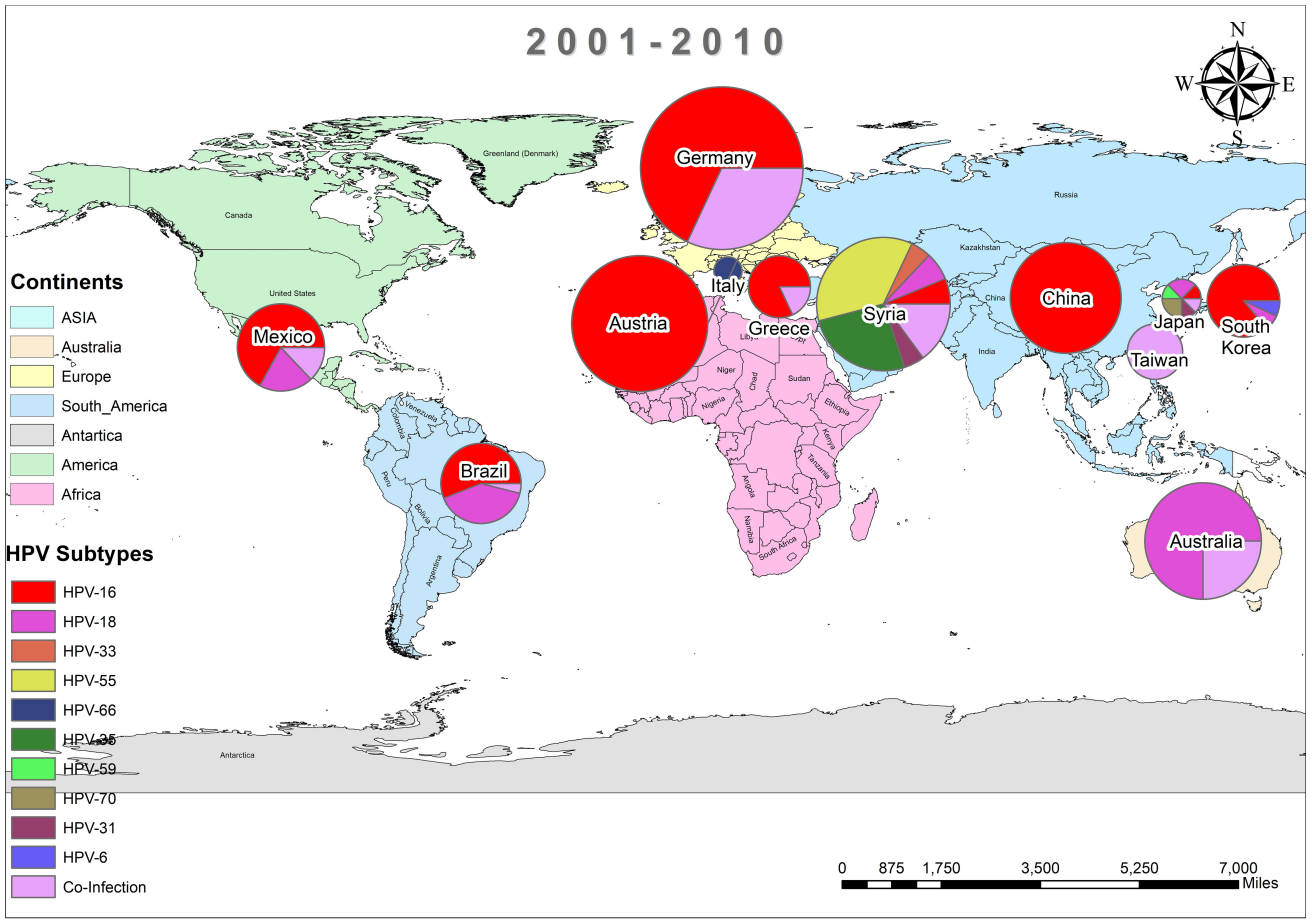
Pool prevalence of HPV in BC patients from 2001-2010.

**Figure 5:**
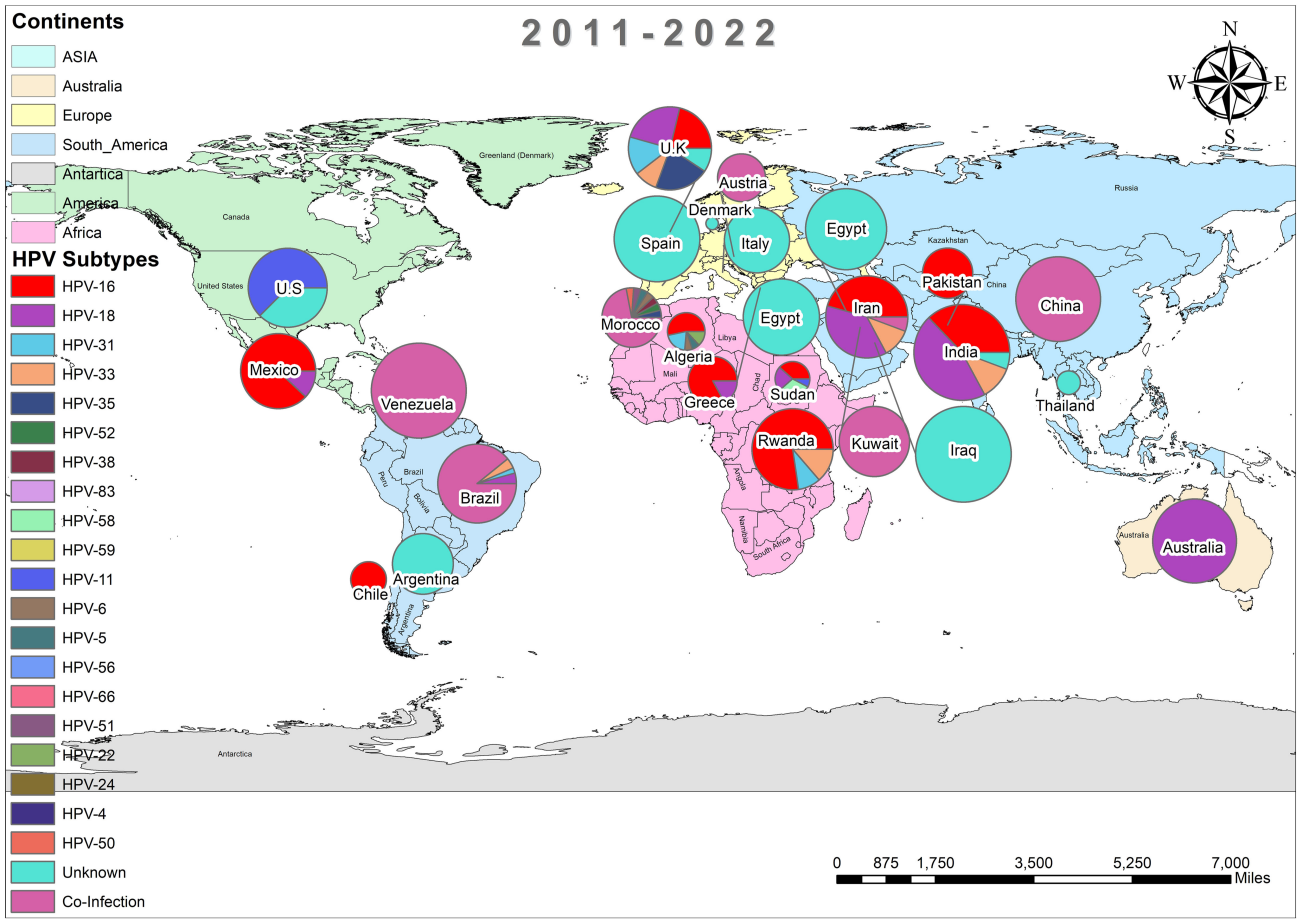
Pool prevalence of HPV in BC patients from 2011-2022.

In addition, the overall prevalence of HPV and the subtypes in individuals diagnosed with BC was calculated for each decade between 1990 and 2022 across all 74 studies. From 1992 to 2000, only three studies reported HPV in breast tissues, primarily subtype HPV-16 and 33, as depicted in **Figure 3** (37, 44, 45). In contrast, a wider range of HPV subtypes was reported in sixteen studies undertaken between 2001-2010, as illustrated in **Figure 4** (1, 39, 46–59). During this time frame, HPV was reported in BC patients in Germany (86%) (48), followed by Austria (64%) (47), Syria (61%) (53), China (60%) (59) and similarly other reported in **Figure 4**. Amongst, the maximum diversity of HPV subtypes was reported in Syria by Akil et al. (12), with the majority of HPV-31 (n=58), followed by HPV-35 (n=39), HPV-18 (n=11), HPV-16 (n=9), and HPV-33 (n=8). Furthermore, between 2011-2022, fifty-six studies reported the occurrence of HPV in BC patients around the globe, as shown in **Figure 5** (10, 30, 31, 60–112). In this decade, the maximum frequency of HPV was reported in Qatar (65%) (110), followed by India (65%) (97), Venezuela (64%) (95), Spain (52%) (92), China (51%) (79), Australia (50%) (61, 64), and remaining are depicted in **Figure 5**.

**Figure 6:**
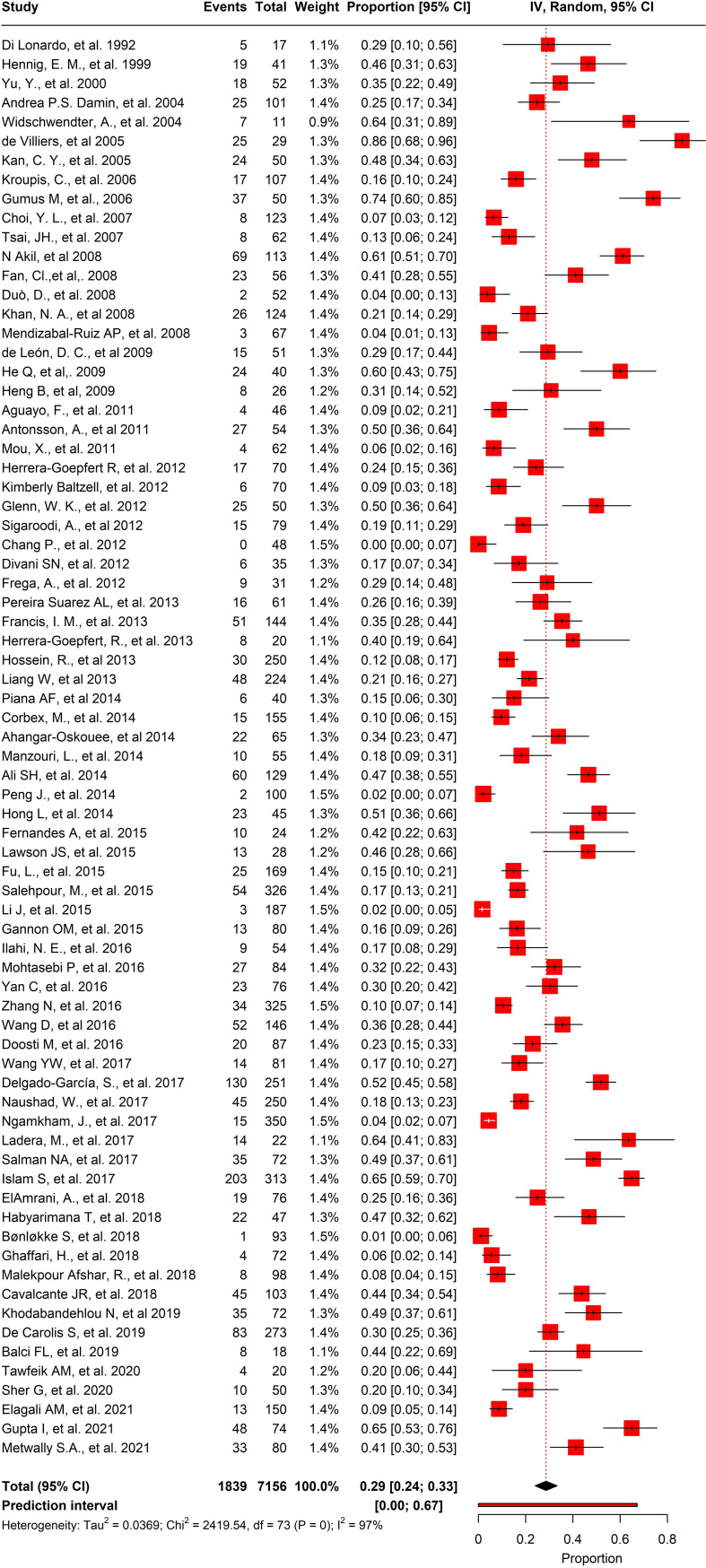
Overall pool prevalence of HPV reported in BC tissues in both case-control and cases-only tissues around the globe from 1992-to-2022.

**Figure 7:**
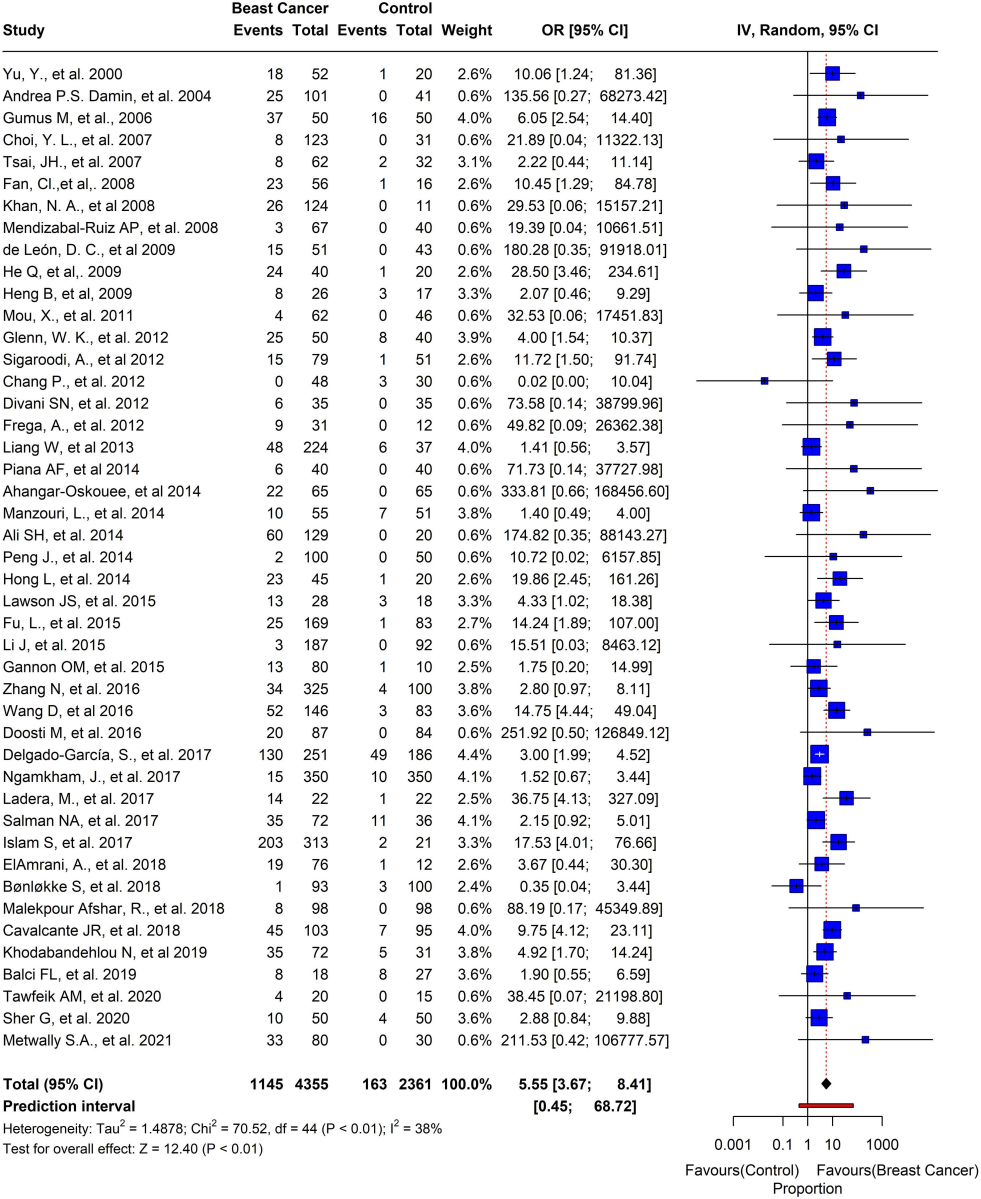
Prevalence of HPV in breast tissues in case-control studies.

Out of the 74 studies we analyzed, the prevalence of HPV in BC was noteworthy. The pooled prevalence showed that 1839 out of 7156 participants tested positive for HPV, with an overall prevalence of 25.6% (95% CI= 0.24-0.33, I2 = 97%, τ^2 = ^0.0369, p = 0) (**Figure 6**). Moreover, we selected 45 studies that had control samples for comparison. The pooled prevalence of HPV in BC samples and control samples was 26.2%, and overall odds of 5.55 (95% CI= 3.67-8.41, I2 = 38%, τ^2 = ^1.4878, p< 0.01) (**Figure 7**).

**Figure 8:**
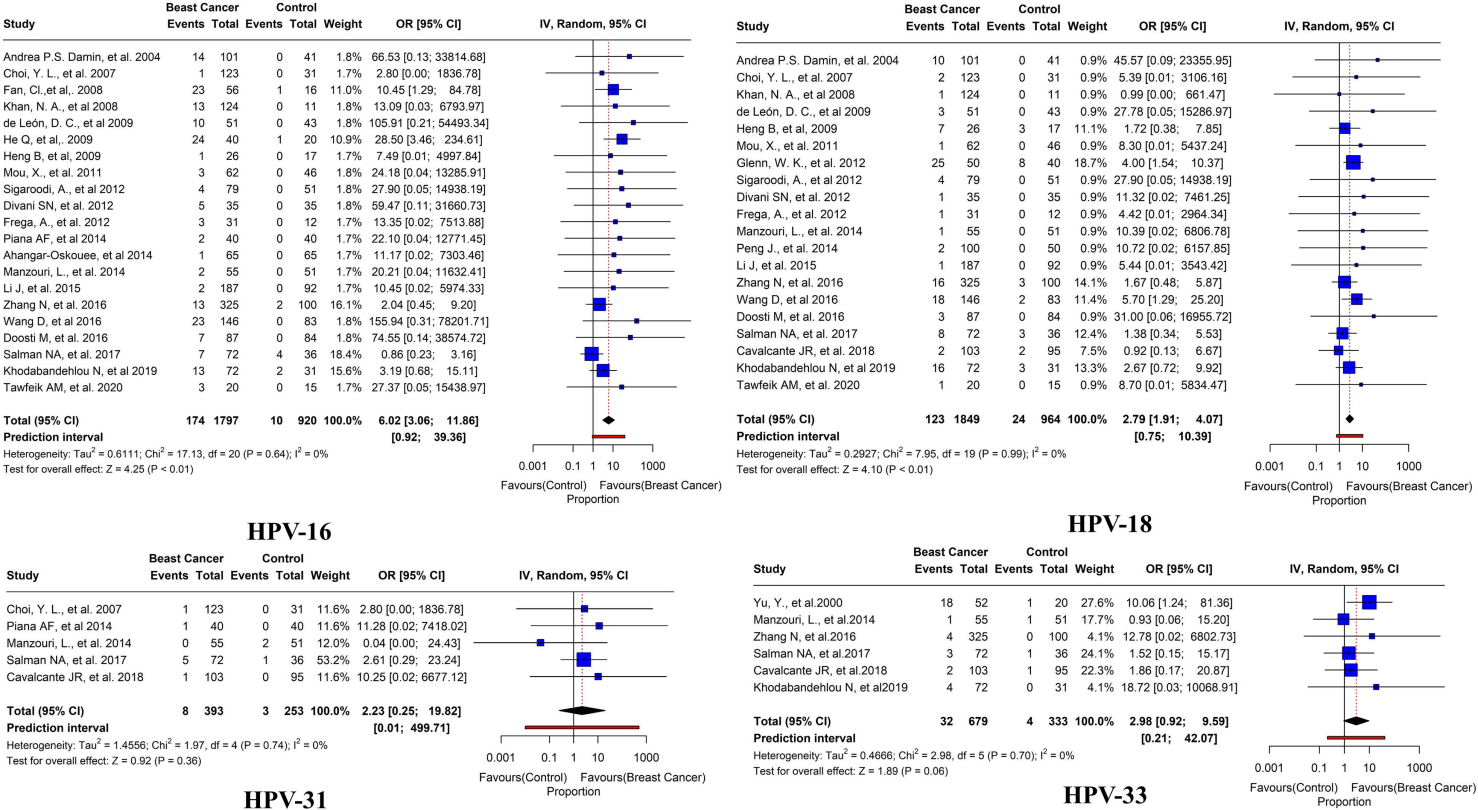
Prevalence of HPV subtypes (16, 18, 31, and 33) in breast tissues in case-control studies.

**Figure 9:**
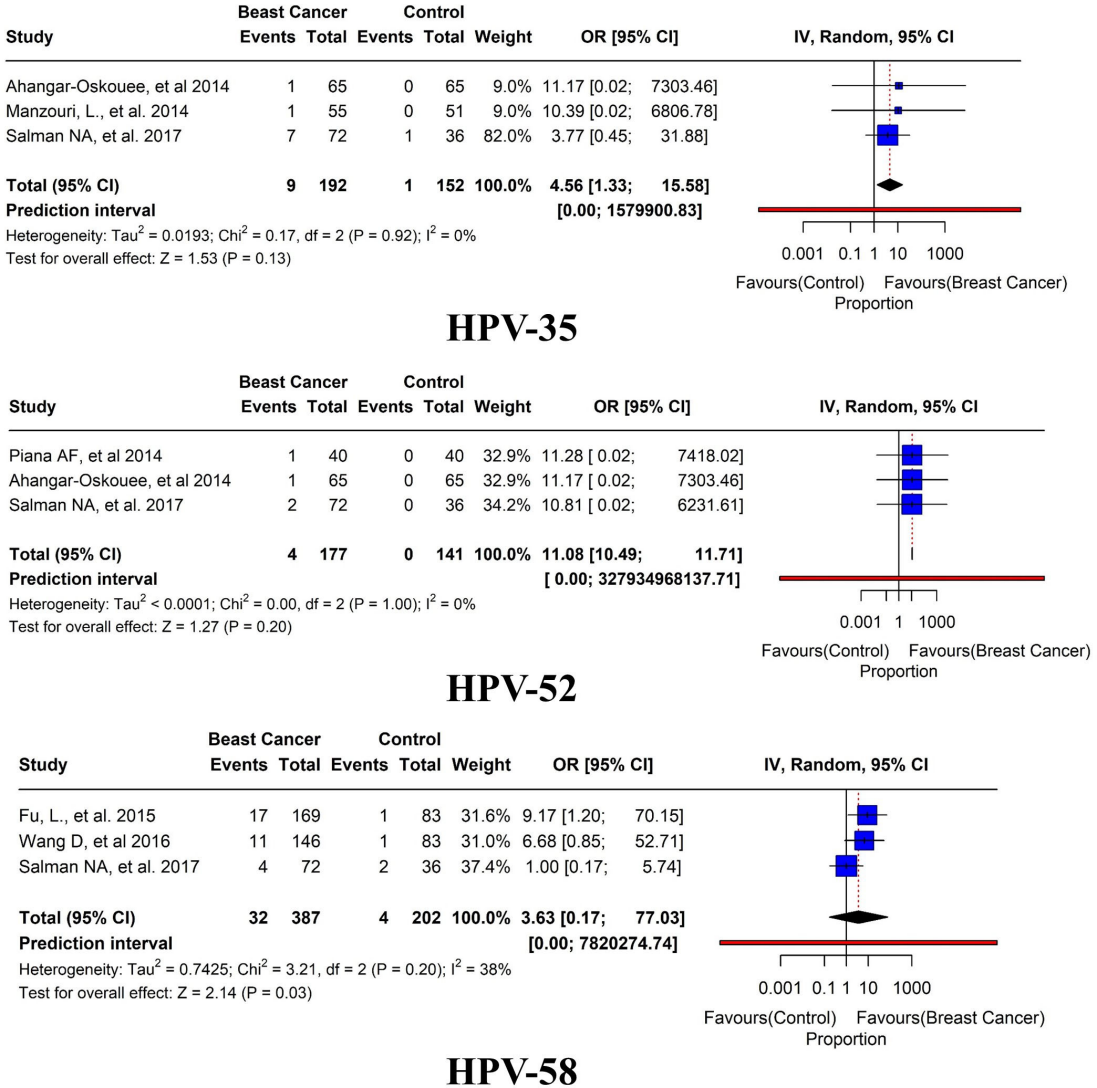
Prevalence of HPV subtypes (35, 52, and 58) in breast tissues in case-control studies.

Of these 45 studies, the further prevalence of HPV subtypes was also studied. HPV-16 was reported in BC tissues in 21 studies with an overall prevalence of 9.6% (95% CI= 3.06-11.86, *I* ^2 = ^0%, τ^2 = ^0.6111, *p*< 0.01). HPV-18 was reported in BC tissues in 20 studies with a prevalence of 6.6% (95% CI=1.91-4.07, *I* ^2 = ^0%, τ^2 = ^0.2927, *p*< 0.01). HPV-31 has been reported in 5 studies with an overall prevalence of 2% (95% CI= 0.25-19.82, *I* ^2 = ^0%, τ^2 = ^1.4556, *p* = 0.36). HPV-33 was reported in 6 studies with an overall prevalence of 4.7% (95% CI= 0.92-9.59, *I* ^2 = ^0%, τ^2 = ^0.4666, *p* = 0.06) (**Figure 8**). HPV 35 was reported in only three studies with an overall prevalence of 4.6% (95% CI=1.33-15.58, *I* ^2 = ^0%, τ^2 = ^0.0193, *p* = 0.13). HPV 52 was reported in only 3 studies with an overall prevalence of 2.3% (95% CI=10.49-11.71, *I* ^2 = ^0%, τ^2^<0.0001, *p* = 0.20). In addition, HPV 58 was reported in only three studies with an overall prevalence of 8.2% (95% CI=0.17-77.03, *I* ^2 = ^38%, τ^2 = ^0.7425, *p* = 0.03) (**Figure 9**).

**Figure 10:**
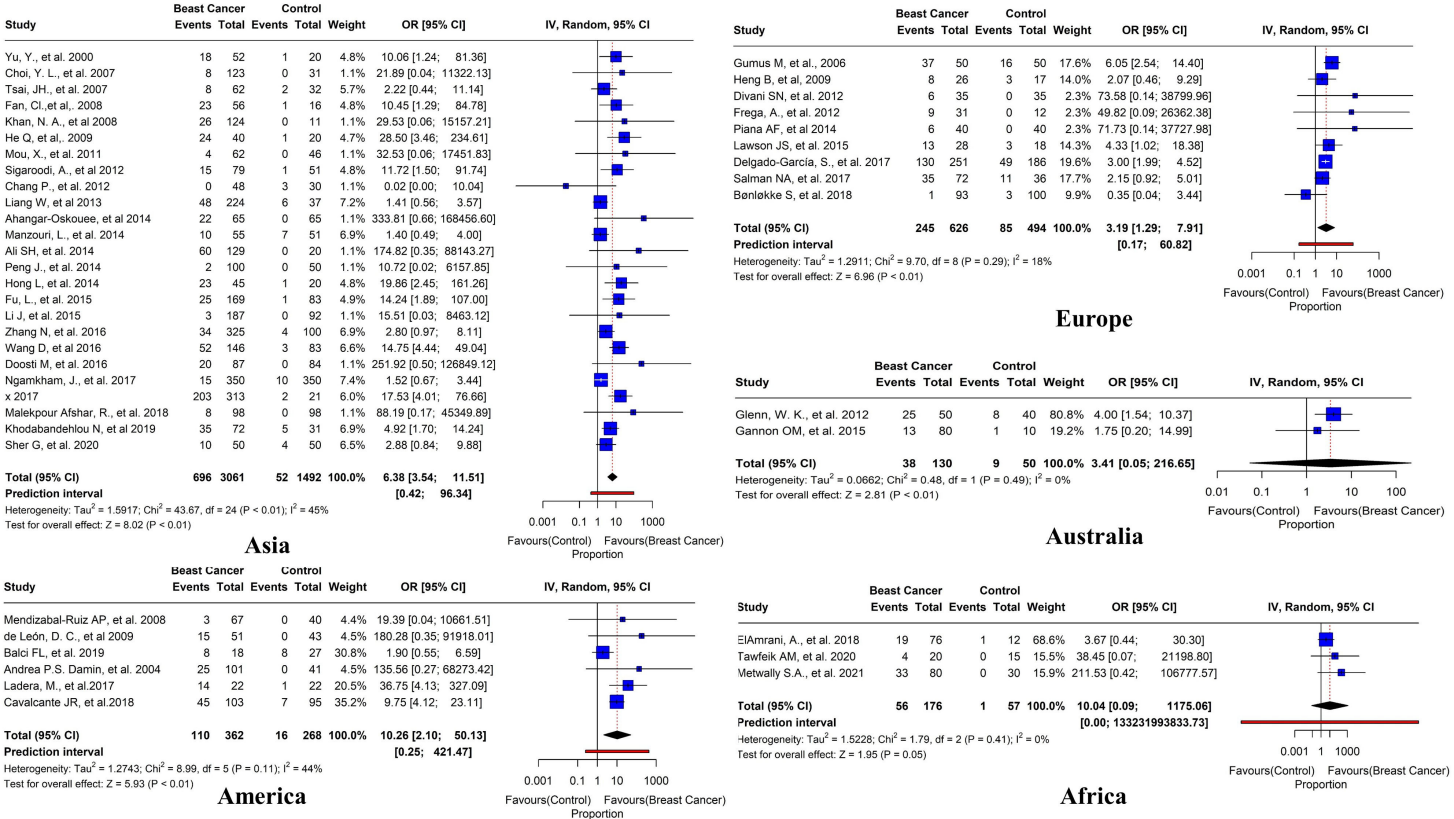
Prevalence of HR HPV subtypes in breast tissues in case-control studies reported in different regions worldwide (Asia, America, Europe, Australia, and Africa).

We conducted a sub-group analysis to examine the regional variations of the prevalence of HPV in BC patients. Surprisingly, Asia had the highest number of studies (n=25) with a pooled prevalence of 22.7% (95% CI=3.54-11.51, *I^2 = ^* 45%, τ2 = 1.5917, p< 0.01). In Europe, nine studies reported positive HPV cases with an overall prevalence of 39.1% (95% CI=1.29-7.91, *I* ^2 = ^18%, τ^2 = ^1.2911, *p*< 0.01). In America, six studies reported positive HPV cases with an overall prevalence of 30.3% (95% CI=2.10-501.13, *I* ^2 = ^44%, τ^2 = ^1.2743, *p*< 0.01). In Australia, two studies reported positive HPV cases with an overall prevalence of 29.2% (95% CI=0.05-216.65, *I* ^2 = ^0%, τ^2 = ^0.0662, *p*< 0.01). Furthermore, in Africa, three studies reported positive HPV cases with an overall prevalence of 31.8% (95% CI=0.09-1175.06, *I* ^2 = ^0%, τ^2 = ^1.5228, *p* = 0.05) (**Figure 10**).

**Figure 11:**
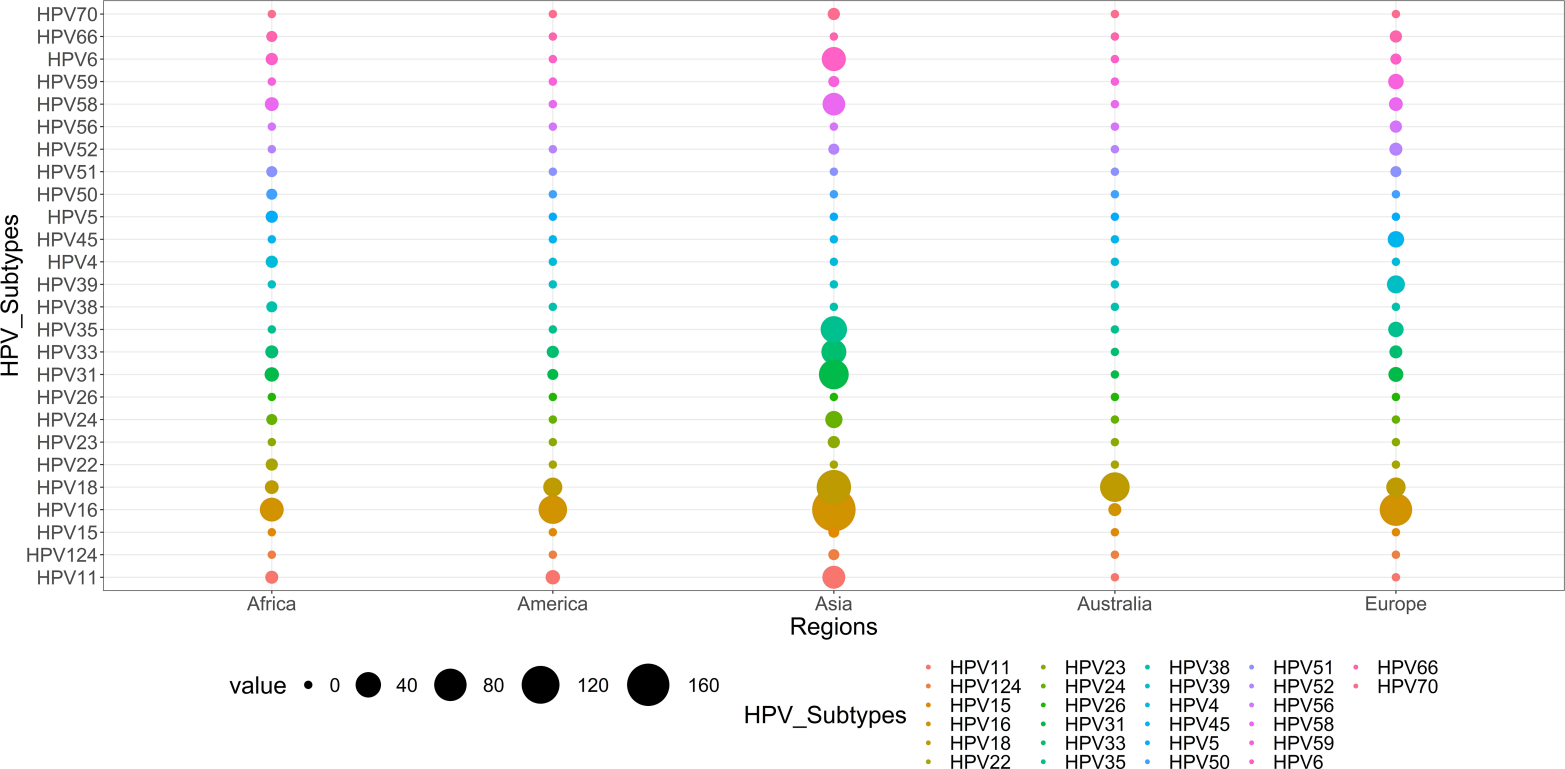
Distribution of HPV subtypes in different continents worldwide.

**Figure 12:**
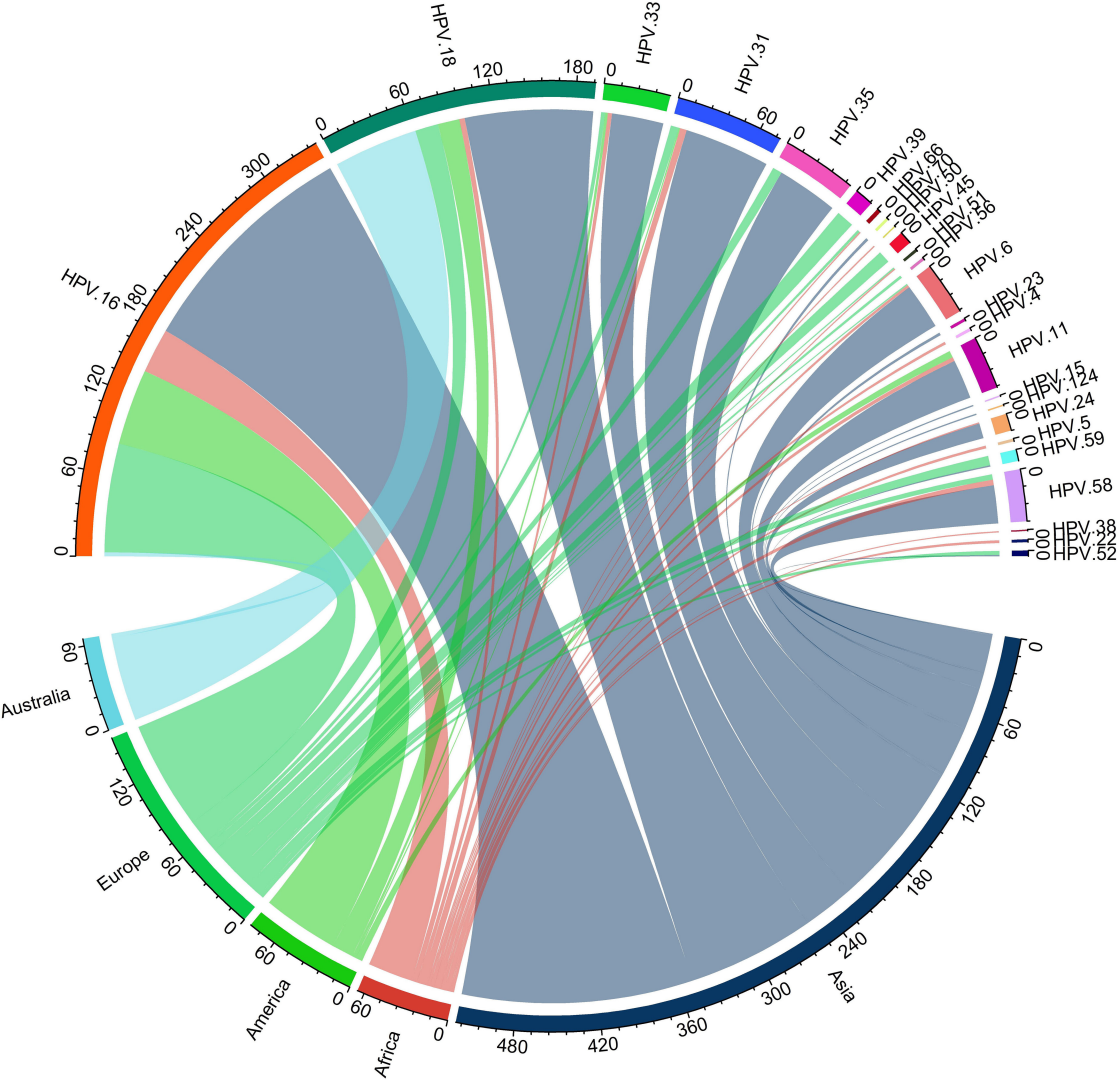
The diversity and distribution of HPV types across five continents. The chords represent the relative frequency of each HPV type, while the colors denote the different continents: Asia, Africa, America, Europe, and Australia.

**Figure 11** illustrates the global diversity of HPV subtypes (n=26) across five continents: Asia, Africa, America, Europe, and Australia. Whereas the distinct color represents each continent, the relative frequency of each HPV subtype is shown by the thickness of the chords, as shown in **Figure 12**. HPV-16 is the dominant subtype in all regions, comprising 342 out of 689 cases. Asia and Europe exhibit the highest HPV-16 cases, with 170 and 80 cases, respectively. Moreover, HPV-18 ranks as the second most common subtype, with 193 cases. The distribution of the other subtypes displays considerable variation across the regions. For example, Australia has an exceptionally high percentage of HPV-18 cases (95.4%); HPV-33 is common in Asia (38) and Africa (3) but scarce in other regions (2 or less). Likewise, HPV-35 is prevalent in Europe (7) and Asia (44) but absent in other regions. Conversely, HPV-31 is largely restricted to Africa (5) and Asia (60), while HPV-45 is mainly limited to Europe (9). The subtypes HPV-23, HPV-4, HPV-26, HPV-15, and HPV-124 in Africa; HPV-70 and HPV-50 in Asia; and HPV-24 and HPV-38 in Europe are extremely rare and only detected in one region.

## 4 Discussion

The causal role of HPV in breast cancer has been a subject of debate for a long time, but recent studies have provided convincing evidence of a positive association between HPV infection and breast cancer development. However, there are still many unresolved questions and challenges in this area of research. Most studies detected HPV using conventional or nested PCR using commercial primers for the L1 gene (capsid protein). After positive results and using primers for E6 and E7 genes or sequencing, they reported probable causes of false positive and false negative results and limitation factors related to the diagnostic techniques used in those studies, like DNA/RNA quality and viral load (113). Approximately 13-15 of the 200 HPV subtypes are HR and associated with various cancers (19). Our comprehensive meta-analysis results revealed that the pooled prevalence of HPV infection among females with BC was 25.6% (95% CI= 0.24-0.33, *I^2 = ^* 97%, τ^2 = ^0.0364, p=0). This finding underscores the urgency of identifying novel risk factors for BC development.

We stratified the control breast tissues and control group to reveal a remarkable finding: a significant difference between breast and control tissues. The pooled prevalence of HPV in BC tissues was 26.2%, with overall odds of 5.55 (95% CI= 3.67-8.41, *I^2 = ^* 38%, τ^2 = ^1.4878, p< 0.01). The *I^2^* value across subgroups was below 50%, indicating acceptable heterogeneity and emphasizing the importance of control selection. Multiple meta-analyses have reported similar results, showing a significant association of HPV with BC. For instance, a meta-analysis of 37 case-control studies containing 3,607 BC cases and 1,728 controls reported overall odds of 6.22 (95% CI = 4.25 to 9.12, p = 0.0002) (114). Another meta-analysis of nine case-control studies reported odds of 5.9 (95% CI = 3.26–10.67) (38). Bae and colleagues performed a meta-analysis of 22 studies and reported odds of 4.02 (95% CI: 2.42–6.68) (2). Similarly, a meta-analysis of ten case–control studies containing 447 BC cases and 275 controls showed an increased breast carcinoma risk with HPV positivity (OR = 3.63, 95% CI = 1.42–9.27) (115). However, the association between HPVs and BC is supported by the consistency of different detection methods of HPVs, which indicate a significantly higher prevalence of HPVs in breast cancer than in control tissues (116).

This review and meta-analysis encompass 3156 articles published over the last three decades. We assessed 74 publications to determine the prevalence of HPV in BC tissues after a rigorous evaluation of 1223 studies, of which 1130 were deemed inadequate. Our findings have been supported by Simoes et al. (38) meta-analyses conducted in Europe, North America, and Australia. According to the studies, HPV infection was more common in Iranian BC patients than in European women but less common in North American and Australian women. Worldwide HPV infection in BC reported up to 86%, indicating disparities between countries may be influenced by demographic factors and geographical differences (2, 38, 117). The conclusion drawn from the previously published figures—approximately one in four women diagnosed with BC have HPV infection (115).

There was a substantial variation in the prevalence of HPV strains observed across various populations (118). HR HPV infections, including HPV 16, 18, and 33, reported as the common causes of genital atypical lesions and cancer, have been identified in BC (56, 119). Intriguingly, HPV 11, 16, 18, and 33 were the most common types in European women with BC, whereas HPV 52, 59, and 83, either HR or LR subtypes, were more prevalent in Asian women (50, 119, 120). In this research, we analyzed HR subtypes of HPV, namely HPV 16, 18, 31, 33, 35, 52, and 58. Our study revealed that all these HR HPVs were associated with an elevated risk of developing BC (*p*<0.05). Our study corroborates the existing literature and suggests that HPV infection may have a causal or contributory effect on BC initiation and progression. The prevalence of HR HPVs in BC tissue is six times higher than in normal and benign breast tissue controls (121). Moreover, HPV-16 was reported in BC tissues in 21 studies with an overall prevalence of 9.7% (95% CI= 3.15-11.73, *I* ^2 = ^0%, τ^2 = ^0.5766, *p<* 0.01). The second most common type found in BC tissues, as reported in 21 studies, was HPV-18 with a prevalence of 6.6% (95% CI=1.95-4.04, *I*2 = 0%, τ^2 = ^0.2734, *p<* 0.01).

In this study, we aimed to investigate the prevalence and distribution of different HPV subtypes in BC tissues from various geographical regions. We found that HPV-16 was the most frequent subtype in Asia, America, Europe, and Africa, while HPV-18 was more prevalent in Australia. A total of 45 studies were examined, with the majority (n=25) published in Asia, indicating a prevalence of (22.7%) overall. We also observed regional variations in the prevalence of HPV among BC patients, with Europe having the highest rate (39.1%), followed by Africa (31.8%), Australia (30.5%), and America (30.3%). Our results suggest that the prevalence of HPV in BC patients varies by location, which may have implications for developing regionally specific methods for preventing and treating BC caused by HPV.

The regional variation of HPV infection and its association with breast cancer may be attributed to multiple factors, such as sexual behavior, hygiene, screening, vaccination, and socioeconomic status. For instance, Asia has a high prevalence of HPV in breast cancer (22.7%) due to its large population of women with multiple sexual partners, low condom use, and limited access to cervical cancer screening and HPV vaccination (122–127). Conversely, Europe has a lower prevalence of HPV in breast cancer (13.4%) due to its more developed health systems, higher awareness of HPV prevention, and higher coverage of HPV vaccination (128–130). Moreover, the oncogenic potential and distribution of HPV subtypes vary across regions (131). For example, HPV 16 and 18, the most common and carcinogenic subtypes of HPV, are more prevalent in breast cancer tissues than other subtypes but are not evenly distributed worldwide (119, 131, 132). Therefore, the regional variation of HPV subtypes may influence the risk of developing breast cancer. Furthermore, the availability and quality of data, the methods of detection, and the selection of samples may affect the estimation of HPV prevalence in breast cancer by country and region (119, 133). However, these results may also be influenced by the heterogeneity of the studies, the sensitivity and specificity of the assays, and the representativeness of the samples (134, 135).

The link between HPV and BC has not been conclusively established, which raises questions about the potential underlying mechanism. The simple detection of HPV is insufficient evidence to prove the virus’s role as a causal agent in the development of BC pathophysiology, and this fact has to be addressed. On the other hand, it is predicted that HPV infection will commence the progression of BC, ultimately resulting in accumulative changes over time similar to the process of cervical carcinogenesis (136). Many hypotheses were offered. Elevated levels of inflammatory cytokines (IL-1, IL-6, IL-17, TGF-, TNF-, and NF-kB) and tumor growth were associated with HPV (137). On the other hand, an essential functional protein, the E6 protein, interacts with p53 and collaborates with BCL2 antagonist/killer (BAK 34) to induce chromosomal instability and apoptotic resistance (138). On the other hand, E7 proteins interact with retinoblastoma (RB) proteins, which triggers the release of E2F (transcription factor), which enhances cellular proliferation. The overexpression of S-phase genes like cyclin A and E is facilitated by E7, which concurrently suppresses cyclin-dependent kinase inhibitors (WAF1/p21) and kinesin-like protein (KIP1/p27) (18, 139). Breast and ovarian cancer susceptibility gene-1 (BRCA1) and BRCA2 are critical biological components linked to HPV proteins. The tumor suppressor activities of these genes are widely recognized for their ability to repair DNA damage and prevent tumor formation, while these proteins activate c-Jun N-terminal kinase/stress-activated protein kinase (JNK/SAPK) to induce apoptosis (140–142). The interaction of E7 and E6 with BRCA1 suppresses several BRCA1-mediated activities (143). In addition, in HPV-induced carcinogenesis, the overexpression of E6 and E7 oncogenes has significant biological importance and role in cancer progression (118, 144, 145). In addition, Michael B. Burns’s study team showed that mutations and deletions in the DNA cytosine deaminase APOBEC3B (A3B), which inhibits retroviral replication, might increase BC risk (146). The team of Vieira and Ohba found out in 2014 that HPV could potentially modify the expression of APOBEC3B (A3B) (147, 148). Hence, it is feasible to hypothesize that HPV may contribute to early BC development by influencing APOBEC3B (A3B) (114, 146).

This systematic review aimed to determine the global prevalence of HPV in BC tissues. In addition, we conducted a meta-analysis of case-control studies, which provided more reliable and informative results than studies that included only positive cases. This study provides a more comprehensive picture of the prevalence patterns of HPV infection by incorporating primary studies from different geographical regions around the globe. It also identifies the most vulnerable communities for targeted interventions.

### 4.1 Limitations

One of the limitations of our study was the inability to determine the association between HPV and BC mortality, as none of the primary studies in our systematic review/meta-analysis addressed this issue. Moreover, we could not examine the effect of coinfections on BC aggressiveness due to insufficient data. To establish the causal role of HPV in BC, it is essential to detect both integrated and free virus DNA, which will improve the methodological quality of HPV detection.

## 5 Conclusion

To estimate the global prevalence of HPV in BC tissues, we performed a meta-analysis of data from multiple studies conducted within the last 30 years. Our findings indicate that HPV infection is a risk factor for developing BC. In addition, we discovered that the prevalence of HPV infection in BC patients is substantially higher than in the general population. Incorporating HPV testing of breast ductal lavage, nasopharyngeal discharge, and breast milk into the cervical screening program is essential for determining breast cancer risk and facilitating early detection. In addition, HPV vaccination may reduce the incidence of breast cancer. However, no research has investigated the link between HPV vaccination and cancer occurrence. Given the heterogeneity and high likelihood of HPV infection in BC lesions, additional rigorous studies employing standardized testing methods are necessary to clarify the role of HPV in BC.

## Data availability statement

The original contributions presented in the study are included in the article/**Supplementary Material**. Further inquiries can be directed to the corresponding authors.

## Author contributions

UA, NA, and AK were involved in the conceptualization, formal analysis, data collection, visualization, and writing the first draft of the manuscript. SA, SKa, UA, SKh, and XG did the data validation, statistical analysis, data interpretation, and figure editing. UA, XG, ZY, and JL were involved in conceptualizing, designing, and supervising the study and did the final revision of the manuscript. All authors listed have made a substantial, direct, and intellectual contribution to the work and approved the final version of the manuscript.
