# Supplementary material for: An updated systemic review and meta-analysis on human papillomavirus in breast carcinogenesis
Source: Front Oncol. 2023 Aug 11;13:1219161. doi: 10.3389/fonc.2023.1219161 (PMC10498127; doi:10.3389/fonc.2023.1219161)
Supplement: Supplementary file 1 [file DataSheet_1.docx]

**Supplementary I Search strategy in PubMed database.**

"HPV"[All Fields] AND ("breast neoplasms"[MeSH Terms] OR ("breast"[All Fields] AND "neoplasms"[All Fields]) OR "breast neoplasms"[All Fields] OR ("breast"[All Fields] AND "neoplasm"[All Fields]) OR "breast neoplasm"[All Fields]) OR "HPV"[All Fields] AND ("breast neoplasms"[MeSH Terms] OR ("breast"[All Fields] AND "neoplasms"[All Fields]) OR "breast neoplasms"[All Fields] OR ("breast"[All Fields] AND "cancer"[All Fields]) OR "breast cancer"[All Fields]) OR ("human papillomavirus viruses"[MeSH Terms] OR ("human"[All Fields] AND "papillomavirus"[All Fields] AND "viruses"[All Fields]) OR "human papillomavirus viruses"[All Fields] OR ("human"[All Fields] AND "papillomavirus"[All Fields]) OR "human papillomavirus"[All Fields]) AND ("breast neoplasms"[MeSH Terms] OR ("breast"[All Fields] AND "neoplasms"[All Fields]) OR "breast neoplasms"[All Fields] OR ("breast"[All Fields] AND "cancer"[All Fields]) OR "breast cancer"[All Fields]) OR ("human papillomavirus viruses"[MeSH Terms] OR ("human"[All Fields] AND "papillomavirus"[All Fields] AND "viruses"[All Fields]) OR "human papillomavirus viruses"[All Fields] OR ("human"[All Fields] AND "papillomavirus"[All Fields]) OR "human papillomavirus"[All Fields]) AND ("breast neoplasms"[MeSH Terms] OR ("breast"[All Fields] AND "neoplasms"[All Fields]) OR "breast neoplasms"[All Fields] OR ("human"[All Fields] AND "mammary"[All Fields] AND "neoplasm"[All Fields])) OR "HPV"[All Fields] AND ("breast"[MeSH Terms] OR "breast"[All Fields] OR "breasts"[All Fields] OR "breast s"[All Fields]) AND ("carcinogenesis"[MeSH Terms] OR "carcinogenesis"[All Fields] OR "carcinogeneses"[All Fields]) OR ("papillomaviridae"[MeSH Terms] OR "papillomaviridae"[All Fields]) AND ("breast"[MeSH Terms] OR "breast"[All Fields] OR "breasts"[All Fields] OR "breast s"[All Fields]) AND ("tissue s"[All Fields] OR "tissues"[MeSH Terms] OR "tissues"[All Fields] OR "tissue"[All Fields]) OR "HPV"[All Fields] AND ("breast neoplasms"[MeSH Terms] OR ("breast"[All Fields] AND "neoplasms"[All Fields]) OR "breast neoplasms"[All Fields] OR ("breast"[All Fields] AND "tumors"[All Fields]) OR "breast tumors"[All Fields]) OR "HPV"[All Fields] AND ("breast neoplasms"[MeSH Terms] OR ("breast"[All Fields] AND "neoplasms"[All Fields]) OR "breast neoplasms"[All Fields] OR ("malignant"[All Fields] AND "tumor"[All Fields] AND "breast"[All Fields]) OR "malignant tumor of breast"[All Fields])
