# Supplementary material for: An updated systemic review and meta-analysis on human papillomavirus in breast carcinogenesis
Source: Front Oncol. 2023 Aug 11;13:1219161. doi: 10.3389/fonc.2023.1219161 (PMC10498127; doi:10.3389/fonc.2023.1219161)
Supplement: Supplementary file 2 [file Table_1.docx]

**Table 1:** Studies reported from 1992-2022 the presence of human papillomavirus in the patients with breast cancer.

| S. No | Studies | Year | Country | HPV-16 | HPV-18 | HPV-33 | HPV-31 | HPV-35 | HPV-39 | HPV-66 | HPV-70 | HPV-50 | HPV-45 | HPV-51 | HPV-56 | HPV-6 | HPV-23 | HPV-4 | HPV-26 | HPV-11 | HPV-15 | HPV-124 | HPV-24 | HPV-5 | HPV-59 | HPV-58 | HPV-38 | HPV-22 | HPV-52 | Co-Infection | Total Cases | Prevalence |
| --- | --- | --- | --- | --- | --- | --- | --- | --- | --- | --- | --- | --- | --- | --- | --- | --- | --- | --- | --- | --- | --- | --- | --- | --- | --- | --- | --- | --- | --- | --- | --- | --- |
| 1 | Di Lonardo, et al.[[44](#_ENREF_44)] | 1992 | Italy | 5 | - | - | - | - | - | - | - | - | - | - | - | - | - | - | - | - | - | - | - | - | - | - | - | - | - | - | 5 | 29% |
| 2 | Hennig, E. M., et al.[[45](#_ENREF_45)] | 1999 | Norway | 19 | - | - | - | - | - | - | - | - | - | - | - | - | - | - | - | - | - | - | - | - | - | - | - | - | - | - | 19 | 46% |
| 3 | Yu, Y., et al.[[46](#_ENREF_46)] | 2000 | China | - | - | 18 | - | - | - | - | - | - | - | - | - | - | - | - | - | - | - | - | - | - | - | - | - | - | - | - | 18 | 35% |
| 4 | Andrea P.S. Damin, et al.[[47](#_ENREF_47)] | 2004 | Brazil | 14 | 10 | - | - | - | - | - | - | - | - | - | - | - | - | - | - | - | - | - | - | - | - | - | - | - | - | 1 | 25 | 25% |
| 5 | Widschwendter, A., et al.[[48](#_ENREF_48)] | 2004 | Austria | 7 | - | - | - | - | - | - | - | - | - | - | - | - | - | - | - | - | - | - | - | - | - | - | - | - | - | - | 7 | 64% |
| 6 | de Villiers, et al[[49](#_ENREF_49)] | 2005 | Germany | 17 | - | - | - | - | - | - | - | - | - | - | - | - | - | - | - | - | - | - | - | - | - | - | - | - | - | 8 | 25 | 86% |
| 7 | Kan, C. Y., et al.[[50](#_ENREF_50)] | 2005 | Australia | - | 18 | - | - | - | - | - | - | - | - | - | - | - | - | - | - | - | - | - | - | - | - | - | - | - | - | 6 | 24 | 48% |
| 8 | Kroupis, C., et al.[[51](#_ENREF_51)] | 2006 | Greece | 14 | - | - | - | - | - | - | - | - | - | - | - | - | - | - | - | - | - | - | - | - | - | - | - | - | - | 3 | 17 | 16% |
| 9 | Gumus M, et al.,[[52](#_ENREF_52)] | 2006 | Germany | - | - | - | - | - | - | - | - | - | - | - | - | - | - | - | - | - | - | - | - | - | - | - | - | - | - | 37 | 37 | 74% |
| 10 | Choi, Y. L., et al.[[53](#_ENREF_53)] | 2007 | South Korea | 1 | 2 | - | 1 | - | - | - | 2 | - | - | - | - | - | - | - | - | - | - | - | - | - | 1 | - | - | - | - | 1 | 8 | 7% |
| 11 | Tsai, JH., et al.[[54](#_ENREF_54)] | 2007 | Taiwan | - | - | - | - | - | - | - | - | - | - | - | - | - | - | - | - | - | - | - | - | - | - | - | - | - | - | 8 | 8 | 13% |
| 12 | N Akil, et al.[[55](#_ENREF_55)] | 2008 | Syria | 9 | 11 | 8 | 58 | 39 | - | - | - | - | - | - | - | - | - | - | - | - | - | - | - | - | - | - | - | - | - | - | 69 | 61% |
| 13 | Fan, Cl., et al.[[56](#_ENREF_56)] | 2008 | China | 23 | - | - | - | - | - | - | - | - | - | - | - | - | - | - | - | - | - | - | - | - | - | - | - | - | - | - | 23 | 41% |
| 14 | Duò, D., et al.[[57](#_ENREF_57)] | 2008 | Italy | - | - | - | - | - | - | 2 | - | - | - | - | - | - | - | - | - | - | - | - | - | - | - | - | - | - | - | - | 2 | 4% |
| 15 | Khan, N. A., et al.[[58](#_ENREF_58)] | 2008 | Japan | 13 | 1 | - | - | - | - | - | - | - | - | - | - | 1 | - | - | - | - | - | - | 11 | - | - | - | - | - | - | - | 26 | 21% |
| 16 | Mendizabal-Ruiz AP, et al.[[59](#_ENREF_59)] | 2008 | Mexico | - | - | - | - | - | - | - | - | - | - | - | - | - | - | - | - | - | - | - | - | - | - | - | - | - | - | 3 | 3 | 4% |
| 17 | de León, D. C., et al.[[60](#_ENREF_60)] | 2009 | Mexico | 10 | 3 | - | - | - | - | - | - | - | - | - | - | - | - | - | - | - | - | - | - | - | - | - | - | - | - | 2 | 15 | 29% |
| 18 | He Q, et al.[[61](#_ENREF_61)] | 2009 | China | 24 | - | - | - | - | - | - | - | - | - | - | - | - | - | - | - | - | - | - | - | - | - | - | - | - | - | - | 24 | 60% |
| 19 | Heng B, et al.[[62](#_ENREF_62)] | 2009 | Australia | 1 | 7 | - | - | - | - | - | - | - | - | - | - | - | - | - | - | - | - | - | - | - | - | - | - | - | - | - | 8 | 31% |
| 20 | Aguayo, F., et al.[[63](#_ENREF_63)] | 2011 | Chile | 4 | - | - | - | - | - | - | - | - | - | - | - | - | - | - | - | - | - | - | - | - | - | - | - | - | - | - | 4 | 9% |
| 21 | Antonsson, A., et al.[[64](#_ENREF_64)] | 2011 | Australia | - | 18 | - | - | - | - | - | - | - | - | - | - | - | - | - | - | - | - | - | - | - | - | - | - | - | - | - | 27 | 50% |
| 22 | Mou, X., et al.[[65](#_ENREF_65)] | 2011 | China | 3 | 1 | - | - | - | - | - | - | - | - | - | - | - | - | - | - | - | - | - | - | - | - | - | - | - | - | - | 4 | 6% |
| 23 | Herrera-Goepfert R, et al.[[66](#_ENREF_66)] | 2012 | Mexico | 15 | - | - | - | - | - | - | - | - | - | - | - | - | - | - | - | - | - | - | - | - | - | - | - | - | - | 2 | 17 | 24% |
| 24 | Kimberly Baltzell, et al.[[67](#_ENREF_67)] | 2012 | Texas | 6 | - | - | - | - | - | - | - | - | - | - | - | - | - | - | - | - | - | - | - | - | - | - | - | - | - | - | 6 | 9% |
| 25 | Glenn, W. K., et al.[[68](#_ENREF_68)] | 2012 | Australia | - | 25 | - | - | - | - | - | - | - | - | - | - | - | - | - | - | - | - | - | - | - | - | - | - | - | - | - | 25 | 50% |
| 26 | Sigaroodi, A., et al.[[70](#_ENREF_70)] | 2012 | Iran | 4 | 4 | - | - | - | - | - | - | - | - | - | - | 2 | 2 | - | - | 1 | 1 | 1 | - | - | - | - | - | - | - | - | 15 | 19% |
| 27 | Chang P., et al.[[71](#_ENREF_71)] | 2012 | China | - | - | - | - | - | - | - | - | - | - | - | - | - | - | - | - | - | - | - | - | - | - | - | - | - | - | - | 0 | 0% |
| 28 | Divani SN, et al.[[72](#_ENREF_72)] | 2012 | Greece | 5 | 1 | - | - | - | - | - | - | - | - | - | - | - | - | - | - | - | - | - | - | - | - | - | - | - | - | - | 6 | 17% |
| 29 | Frega, A., et al.[[73](#_ENREF_73)] | 2012 | Italy | 3 | 1 | - | - | - | - | - | - | - | - | 1 | 1 | 1 | - | - | - | - | - | - | - | - | - | - | - | - | - | 2 | 9 | 29% |
| 30 | Pereira Suarez AL, et al.[[74](#_ENREF_74)] | 2013 | Argentina | - | - | - | - | - | - | - | - | - | - | - | - | - | - | - | - | - | - | - | - | - | - | - | - | - | - | 16 | 16 | 26% |
| 31 | Francis, I. M., et al.[[75](#_ENREF_75)] | 2013 | Kuwait | - | - | - | - | - | - | - | - | - | - | - | - | - | - | - | - | - | - | - | - | - | - | - | - | - |  | 51 | 51 | 35% |
| 32 | Herrera-Goepfert, R., et al.[[76](#_ENREF_76)] | 2013 | Mexico | 7 | 1 | - | - | - | - | - | - | - | - | - | - | - | - | - | - | - | - | - | - | - | - | - | - | - | - | - | 8 | 40% |
| 33 | Hossein, R., et al.[[77](#_ENREF_77)] | 2013 | Iran | 13 | 9 | 2 | 3 | 2 | - | - | - | - | - | - | - | - | - | - | - | 1 | - | - | - | - | - | - | - | - | - | - | 30 | 12% |
| 34 | Liang W, et al.[[78](#_ENREF_78)] | 2013 | China | - | - | - | - | - | - | - | - | - | - | - | - | - | - | - | - | - | - | - | - | - | - | - | - | - | - | 48 | 48 | 21% |
| 35 | Piana AF, et al.[[79](#_ENREF_79)] | 2014 | Italy | 2 |  |  | 1 |  |  |  |  |  | 1 |  |  |  |  |  |  |  |  |  |  |  |  |  |  |  | 1 | 1 | 6 | 15% |
| 36 | Corbex, M., et al.[[80](#_ENREF_80)] | 2014 | Algeria | 8 | - | - | 3 | - | - | - | - | - | - | - | - | 1 | - | - | - | - | - | - | - | 1 | - | - | - | 2 | - | - | 15 | 10% |
| 37 | Ahangar-Oskouee, et al.[[81](#_ENREF_81)] | 2014 | Iran | 1 | - | - | - | 1 | - | - | - | - | - | - | - | 17 | - | - | - | 1 | - | - | - | - | - | - | - | - | 1 | 1 | 22 | 34% |
| 38 | Manzouri, L., et al.[[82](#_ENREF_82)] | 2014 | Iran | 2 | 1 | 1 | - | 1 | - | - | - | - | - | - | - | - | - | - | - | 2 | - | - | - | - | - | - | - | - | - | 6 | 10 | 18% |
| 39 | Ali SH, et al.[[83](#_ENREF_83)] | 2014 | Iraq | - | - | - | - | - | - | - | - | - | - | - | - | - | - | - | - | - | - | - | - | - | - | - | - | - |  | 60 | 60 | 47% |
| 40 | Peng J., et al.[[84](#_ENREF_84)] | 2014 | China | - | 2 | - | - | - | - | - | - | - | - | - | - | - | - | - | - | - | - | - | - | - | - | - | - | - | - | - | 2 | 2% |
| 41 | Hong L, et al.[[85](#_ENREF_85)] | 2014 | China | - | - | - | - | - | - | - | - | - | - | - | - | - | - | - | - | - | - | - | - | - | - | - | - | - | - | 23 | 23 | 51% |
| 42 | Gannon OM, et al.[[86](#_ENREF_86)] | 2015 | Australia | - | - | - | - | - | - | - | - | - | - | - | - | - | - | - | - | - | - | - | - | - | - | - | - | - | - | 13 | 13 | 17% |
| 43 | Fernandes A, et al.[[87](#_ENREF_87)] | 2015 | Venezuela | - | - | - | - | - | - | - | - | - | - | - | - | - | - | - | - | - | - | - | - | - | - | - | - | - | - | 10 | 10 | 42% |
| 44 | Lawson JS, et al.[[88](#_ENREF_88)] | 2015 | Australia | - | - | - | - | - | - | - | - | - | - | - | - | - | - | - | - | - | - | - | - | - | - | - | - | - | - | 13 | 13 | 46% |
| 45 | Fu, L., et al.[[89](#_ENREF_89)] | 2015 | Shaanxi | - | - | - | - | - | - | - | - | - | - | - | - | - | - | - | - | - | - | - | - | - | - | 17 | - | - | - | 8 | 25 | 15% |
| 46 | Salehpour, M., et al.[[90](#_ENREF_90)] | 2015 | Iran | - | - | - | - | - | - | - | - | - | - | - | - | 5 | - | - | - | 19 | - | - | - | - | - | - | - | - | - | 30 | 54 | 17% |
| 47 | Li J, et al.[[10](#_ENREF_10)] | 2015 | China | 2 | 1 | - | - | - | - | - | - | - | - | - | - | - | - | - | - | - | - | - | - | - | - | - | - | - | - | - | 3 | 2% |
| 48 | Ilahi, N. E., et al.[[91](#_ENREF_91)] | 2016 | Pakistan | 9 | - | - | - | - | - | - | - | - | - | - | - | - | - | - | - | - | - | - | - | - | - | - | - | - | - | - | 9 | 17% |
| 49 | Mohtasebi P, et al.[[92](#_ENREF_92)] | 2016 | Iran | 10 | 9 | 1 | 1 | 2 | - | - | - | - | - | - | - | - | - | - | - | 4 | - | - | - | - | - | - | - | - | - | - | 27 | 32% |
| 50 | Yan C, et al.[[93](#_ENREF_93)] | 2016 | China | - | - | - | - | - | - | - | - | - | - | - | - | - | - | - | - | - | - | - | - | - | - | - | - | - | - | 23 | 23 | 30% |
| 51 | Zhang N, et al.[[94](#_ENREF_94)] | 2016 | China | 13 | 16 | 4 | - | - | - | - | - | - | - | - | - | - | - | - | - | - | - | - | - | - | - | - | - | - | - | 1 | 34 | 10% |
| 52 | Wang D, et al.[[95](#_ENREF_95)] | 2016 | China | 23 | 18 | - | - | - | - | - | - | - | - | - | - | - | - | - | - | - | - | - | - | - | - | 11 | - | - | - | 7 | 52 | 36% |
| 53 | Doosti M, et al.[[96](#_ENREF_96)] | 2016 | Iran | 7 | 3 | - | - | - | - | - | - | - | - | - | - | 9 | - | - | - | 1 | - | - | - | - | - | - | - | - | - | - | 20 | 23% |
| 54 | Wang YW, et al.[[97](#_ENREF_97)] | 2017 | China | - | - | - | - | - | - | - | - | - | - | - | - | - | - | - | - | - | - | - | - | - | - | - | - | - | - | 14 | 14 | 17% |
| 55 | Delgado-García, S., et al.[[98](#_ENREF_98)] | 2017 | Spain | - | - | - | - | - | - | - | - | - | - | - | - | - | - | - | - | - | - | - | - | - | - | - | - | - | - | 130 | 130 | 52% |
| 56 | Naushad, W., et al.[[99](#_ENREF_99)] | 2017 | Pakistan | - | - | - | - | - | - | - | - | - | - | - | - | - | - | - | - | - | - | - | - | - | - | - | - | - | - | 45 | 45 | 18% |
| 57 | Ngamkham, J., et al.[[100](#_ENREF_100)] | 2017 | Thailand | - | - | - | - | - | - | - | - | - | - | - | - | - | - | - | - | - | - | - | - | - | - | - | - | - | - | 15 | 15 | 4% |
| 58 | Ladera, M., et al.[[101](#_ENREF_101)] | 2017 | Venezuela | - | - | - | - | - | - | - | - | - | - | - | - | - | - | - | - | - | - | - | - | - | - | - | - | - | - | 14 | 14 | 64% |
| 59 | Salman NA, et al.[[102](#_ENREF_102)] | 2017 | UK | 7 | 8 | 3 | 5 | 7 | 13 | - | - | - | 8 | - | 1 | - | - | - | - | - | - | - | - | - | 7 | 4 | - | - | 2 | - | 35 | 49% |
| 60 | Islam S, et al.[[103](#_ENREF_103)] | 2017 | India | - | - | - | - | - | - | - | - | - | - | - | - | - | - | - | - | - | - | - | - | - | - | - | - | - | - | 203 | 203 | 65% |
| 61 | ElAmrani, A., et al.[[104](#_ENREF_104)] | 2018 | Morocco | - | - | - | - | - | - | 1 | - | 1 | - | 1 | - | 1 | - | 2 | - | 2 | - | - | 1 | 1 | - | - | 1 | - | - | 8 | 19 | 25% |
| 62 | Habyarimana T, et al.[[105](#_ENREF_105)] | 2018 | Rwanda | 17 | - | 3 | 2 | - | - | - | - | - | - | - | - | - | - | - | - | - | - | - | - | - | - | - | - | - | - | - | 22 | 47% |
| 63 | Bønløkke S, et al.[[106](#_ENREF_106)] | 2018 | Denmark | - | - | - | - | - | - | - | - | - | - | - | - | - | - | - | - | - | - | - | - | - | - | - | - | - | - | 1 | 1 | 1% |
| 64 | Ghaffari, H., et al.[[107](#_ENREF_107)] | 2018 | Iran | - | - | - | - | - | - | - | - | - | - | - | - | - | - | - | - | - | - | - | - | - | - | - | - | - | - | 4 | 4 | 6% |
| 65 | Malekpour Afshar, R., et al.[[108](#_ENREF_108)] | 2018 | Iran | - | - | - | - | - | - | - | - | - | - | - | - | - | - | - | - | - | - | - | - | - | - | - | - | - | - | 8 | 8 | 8% |
| 66 | Cavalcante JR, et al.[[109](#_ENREF_109)] | 2018 | Brazil | - | 2 | 2 | 1 | - | - | - | - | - | - | - | - | - | - | - | - | - | - | - | - | - | - | - | - | - | - | 37 | 45 | 44% |
| 67 | Khodabandehlou N, et al.[[110](#_ENREF_110)] | 2019 | Iran | 13 | 16 | 4 | - | - | - | - | - | - | - | - | - | - | - | - | - | - | - | - | - | - | - | - | - | - | - | 2 | 35 | 49% |
| 68 | De Carolis S, et al.[[111](#_ENREF_111)] | 2019 | Italy | - | - | - | - | - | - | - | - | - | - | - | - | - | - | - | - | - | - | - | - | - | - | - | - | - | - | 83 | 83 | 30% |
| 69 | Balci FL, et al.[[112](#_ENREF_112)] | 2019 | United States | - | - | - | - | - | - | - | - | - | - | - | - | - | - | - | 5 | - | - | - | - | - | - | - | - | - | - | 3 | 8 | 44% |
| 70 | Tawfeik AM, et al.[[113](#_ENREF_113)] | 2020 | Egypt | 3 | 1 | - | - | - | - | - | - | - | - | - | - | - | - | - | - | - | - | - | - | - | - | - | - | - | - | - | 4 | 20% |
| 71 | Sher G, et al.[[114](#_ENREF_114)] | 2020 | Qatar | - | - | - | - | - | - | - | - | - | - | - | - | - | - | - | - | - | - | - | - | - | - | - | - | - | - | 10 | 10 | 20% |
| 72 | Elagali AM, et al.[[115](#_ENREF_115)] | 2021 | Sudan | 5 | 3 | - | - | - | - | - | - | - | - | - | - | - | - | - | - | 1 | - | - | - | - | - | 4 | - | - | - | - | 13 | 9% |
| 73 | Gupta I, et al.[[116](#_ENREF_116)] | 2021 | Qatar | - | - | - | - | - | - | - | - | - | - | - | - | - | - | - | - | - | - | - | - | - | - | - | - | - | - | 48 | 48 | 65% |
| 74 | Metwally S.A., et al.[[117](#_ENREF_117)] | 2021 | Egypt | - | - | - | - | - | - | - | - | - | - | - | - | - | - | - | - | - | - | - | - | - | - | - | - | - | - | 33 | 33 | 41% |

*Note: “-” subtype not reported; HPV (human Papillomavirus)*
